# Neoadjuvant chemotherapy versus surgery alone in locally advanced esophageal squamous cell carcinoma: a propensity-matched real-world study

**DOI:** 10.3389/fonc.2025.1691998

**Published:** 2025-11-24

**Authors:** Yuchen Wang, Zhifeng Yue, Zhifeng Li, Jin Yang, Jifang Yao, Junfeng Liu

**Affiliations:** 1 Department of Thoracic Surgery, The Fourth Hospital of Hebei Medical University, Shijiazhuang, Hebei, China; 2 Department of Anesthesiology, The Fourth Hospital of Hebei Medical University, Shijiazhuang, Hebei, China

**Keywords:** esophageal squamous cell carcinoma, pathological complete response, neoadjuvant chemotherapy, survival benefits, survival outcomes

## Abstract

**Background:**

The therapeutic value of neoadjuvant chemotherapy (NAC) for locally advanced esophageal squamous cell carcinoma (ESCC) remains uncertain compared to surgery alone (SA), particularly in real-world settings. This study utilized propensity score matching (PSM) to compare survival outcomes between NAC and surgery in a well-balanced ESCC cohort, while assessing pathological complete response (pCR) and prognostic factors to guide clinical decision-making.

**Methods:**

The study conducted a retrospective analysis of 690 patients with locally advanced ESCC (T3-4aN0-3M0) who underwent radical esophagectomy between 2009 and 2019. PSM was employed to balance baseline characteristics, yielding 452 matched patients (135 NAC, 317 SA). NAC consisted of platinum-based doublet regimens. Survival outcomes, including disease-free survival (DFS) and overall survival (OS), were analyzed using Kaplan-Meier and Cox regression methods. pCR were assessed using CAP criteria.

**Results:**

NAC significantly improved 5-year DFS (28.7% vs. 18.5%, *P* = 0.001) and OS (37.9% vs. 24.2%, *P* = 0.001) compared to SA. Patients achieving pCR (11.9%) exhibited superior DFS (5-year: 55.0% vs. 18.5%, *P* = 0.010) and OS (5-year: 59.8% vs. 35.0%, *P* = 0.019). Multivariate analysis identified NAC, histologic grade, ypN stage, vessel/nerve invasion, and pCR as independent prognostic factors.

**Conclusions:**

This real-world data supports that NAC significantly improves survival outcomes compared to SA in locally advanced ESCC. pCR post-NAC independently predicted improved OS.

## 1 Introduction

Esophageal squamous cell carcinoma (ESCC) remains one of the most aggressive malignancies worldwide, with locally advanced disease presenting particularly poor prognosis (1, 2). While surgical resection forms the cornerstone of treatment, the 5-year survival rates for surgery alone remain dismal, rarely exceeding 25% (3). This sobering reality has driven the development of multimodal approaches, with neoadjuvant chemoradiotherapy (NCRT) emerging as the current standard based on landmark trials including CROSS (4) and NEOCRTEC 5010 (5).

While the landmark CROSS and NEOCRTEC 5010 trials established NCRT as the standard of care for locally advanced ESCC, demonstrating significant survival benefits. However, most of these trials and studies have focused on data from esophageal cancer patients in Western countries, where the predominant pathological type is adenocarcinoma. Thus, their guidance for the treatment of ESCC is limited. In addition, a study by Ken Kato et al. (6) based on an Asian population with ESCC showed that, compared to neoadjuvant chemotherapy (NAC), NCRT did not significantly improve patient survival rates. Moreover, the incidence of treatment-related adverse events and postoperative complications was higher with NCRT than with NAC (7, 8). On the other hand, the role of NAC remains controversial and less well-defined. While some studies suggest comparable efficacy between NAC and NCRT (6–10), others report inferior outcomes with chemotherapy alone (7). This controversy stems from several critical knowledge gaps: (1) limited real-world evidence comparing NAC directly with SA in rigorously matched cohorts, (2) uncertainty regarding pathological complete response (pCR) rates and their prognostic significance in NAC-treated ESCC, and (3) inadequate understanding of predictive biomarkers for chemotherapy response.

To address these gaps, we conducted a large-scale real-world study to retrospectively analyze patients with locally advanced ESCC (T3-4aN0-3M0) who underwent radical esophagectomy at our center. Using propensity score matching (PSM) analysis of the enrolled cohort, performed Kaplan-Meier survival analysis and multivariate Cox regression to identify prognostic factors influencing outcomes in ESCC patients receiving neoadjuvant chemotherapy. These findings provide an evidence-based theoretical foundation for optimizing personalized treatment strategies for this patient population.

## 2 Methods

### 2.1 Study design and patient selection

This study was a real-world retrospective analysis with PSM to investigate the survival outcomes of patients with locally advanced ESCC who received platinum-based doublet chemotherapy as neoadjuvant treatment at The Fourth Hospital of Hebei Medical University between January 2009 and January 2019. Eligible patients met the following criteria: (1) underwent radical esophagectomy; (2) tumor located in the thoracic esophagus; (3) histologically confirmed squamous cell carcinoma; (4) clinical stage (neoadjuvant chemotherapy group) or pathological stage (surgery-alone group) classified as T3–4aN0–3M0 according to the 8th edition of the American Joint Committee on Cancer (AJCC) staging system for esophageal cancer (11); (5) complete clinical data available. Patients were excluded if they had (1) perioperative mortality; (2) patients who received any additional anticancer therapy (such as radiotherapy or immunotherapy) during or after the neoadjuvant chemotherapy; (3) received fewer than 2 cycles of neoadjuvant chemotherapy; (4) intraoperative lymph node dissection with fewer than 15 nodes harvested.

Diagnosis and clinical staging were determined using contrast-enhanced computed tomography (CT) of the chest and abdomen, contrast-enhanced magnetic resonance imaging (MRI), endoscopic ultrasonography (EUS), and cervical ultrasonography. Positron emission tomography-computed tomography (PET-CT) was additionally performed of necessary. The pathological response to neoadjuvant chemotherapy was evaluated according to the College of American Pathologists (CAP) tumor regression grading system (12).

The study was approved by the Ethics Committee of The Fourth Hospital of Hebei Medical University (NO.2022MECD58) and was strictly conducted in accordance with the Declaration of Helsinki (as revised 2013).

### 2.2 Neoadjuvant chemotherapy before radical resection of esophageal cancer

The neoadjuvant chemotherapy regimens were administered according to contemporary NCCN and CSCO guidelines. According to the drug instructions, patients underwent neoadjuvant chemotherapy with a platinum-based doublet regimen consisting of paclitaxel (175 mg/m² intravenously on day 1) combined with either carboplatin (300 mg/m² intravenously on day 2) or cisplatin (15 mg/m²/day intravenously on days 1-5), administered in 3–4 week cycles. The treatment comprised 2–4 cycles, followed by surgical resection 4–6 weeks after completion of chemotherapy.

### 2.3 Surgical procedure for radical resection of esophageal cancer

All patients underwent standardized radical esophagectomy using either the Ivor-Lewis approach (open or minimally invasive) with right thoracic esophagogastric anastomosis or the McKeown approach (open or minimally invasive) with cervical anastomosis.

### 2.4 Follow-up

The follow-up information was obtained through our hospital’s follow-up center by means of phone calls, letters, and review of inpatient and outpatient records. The follow-up started from the date of the surgery and was calculated monthly until the last follow-up or the date of the clinical event occurred. The follow-up deadline was December 2024. Patients who failed to respond to two consecutive follow-up reminders were defined as lost to follow-up. In the survival analysis, these patients were censored at the date of the first missed follow-up. The follow-up content for postoperative patients included clinical symptoms, signs, and results of various examinations and tests. The examinations included gastrointestinal radiography, gastroscopy, computed tomography (CT) (head, chest and abdominal), ultrasound, etc. Hematological tests included tumor markers, etc. If necessary, tissue pathological biopsy was performed to determine whether there was recurrence and/or metastasis. Disease-free survival (DFS) was defined as the time from surgery to disease recurrence. Local or lymph node recurrence and metastatic diseases were considered as indications of recurrence of the primary tumor. Overall survival (OS) was defined as the time from surgery to death due to any cause, and the longest follow-up time was used as the termination criterion.

### 2.5 Statistical analysis

All statistical analyses were performed with SPSS 22.0. Categorical variables were summarized as frequencies with percentages. For survival analysis, Kaplan-Meier estimates were generated and compared using log-rank tests in univariate analysis. Variables demonstrating significant associations (*P* < 0.05) in univariate analysis were subsequently included in multivariate Cox proportional hazards regression models with forward selection to identify independent prognostic factors for survival outcomes. All *P* < 0.05 was considered significant.

## 3 Results

### 3.1 Basic characteristics of participants

Between January 2009 and January 2019, 703 patients with locally advanced ESCC who underwent surgical resection at our center were initially reviewed. After applying inclusion and exclusion criteria, 690 eligible patients with complete clinical and follow-up data were included in this study. Of these, 138 patients received neoadjuvant chemotherapy (NAC) followed by surgery, while 552 underwent surgery alone (SA). The baseline characteristics of the entire cohort are summarized in **Table 1**.

**Table 1:** Basic characteristics of participants.

Characteristics	Before PSM		χ^2^	*P*	After PSM		χ^2^	*P*
	NAC (n=138)	SA(n=552)			NAC (n=135)	SA (n=317)		
Gender								
Male	93	417	3.805	0.051	90	226	0.963	0.326
Female	45	135			45	91		
Age (years)								
≤60	76	307	0.013	0.909	75	176	<0.001	0.995
>60	26	245			60	141		
Smoking								
Yes	60	281	2.437	0.119	57	165	3.659	0.056
No	78	271			78	152		
Alcohol Drinking								
Yes	52	247	2.244	0.134	52	144	2.258	0.133
No	86	305			86	173		
ECOG PS Score								
0	68	307	2.625	0.269	67	175	1.256	0.534
1	57	210			56	119		
2	13	35			12	23		
Location								
Upper	7	55	35.636	<0.001	7	39	14.924	0.001
Middle	73	147			71	109		
Lower	58	350			57	169		
Primary Tumor								
T3	124	516	2.156	0.142	124	299	0.962	0.327
T4	14	36			11	18		
Regional Lymph Nodes								
N0	66	300	3.276	0.351	66	171	2.085	0.555
N1	40	155			39	83		
N2	24	78			24	44		
N3	8	19			6	19		
Histologic Grade								
G1	1	4	12.124	0.002	1	3	1.659	0.391
G2	83	241			80	167		
G3	54	307			54	147		
Surgical Approaches								
OE	120	465	0.632	0.427	117	286	1.237	0.266
MIE	18	87			18	31		
Vessel or nerve invasion								
Positive	10	15	6.485	0.011	9	10	2.900	0.089
Negative	128	537			126	307		

PSM, Propensity Score Matching; NCT, Neoadjuvant chemotherapy; SA, Surgery alone; ECOG PS score, Eastern Cooperative Oncology Group performance status score; OE, Open esophagectomy; MIE, Minimally invasive esophagectomy.

To mitigate potential selection bias, we performed a one-to-three PSM analysis (nearest-neighbor matching, caliper=0.1) using the NAC group as the reference. Matching variables included tumor location, regional lymph nodes status, clinical T stage, histologic grade, surgical approach,vessel or nerve invasion. After PSM, the final analysis included 452 patients, 135 patients in the NAC group and 317 patients in the SA group, respectively. The baseline clinical characteristics were well balanced after PSM between two groups (**Table 1**).

### 3.2 Evaluation of disease-free survival and overall survival

**Figure 1:**
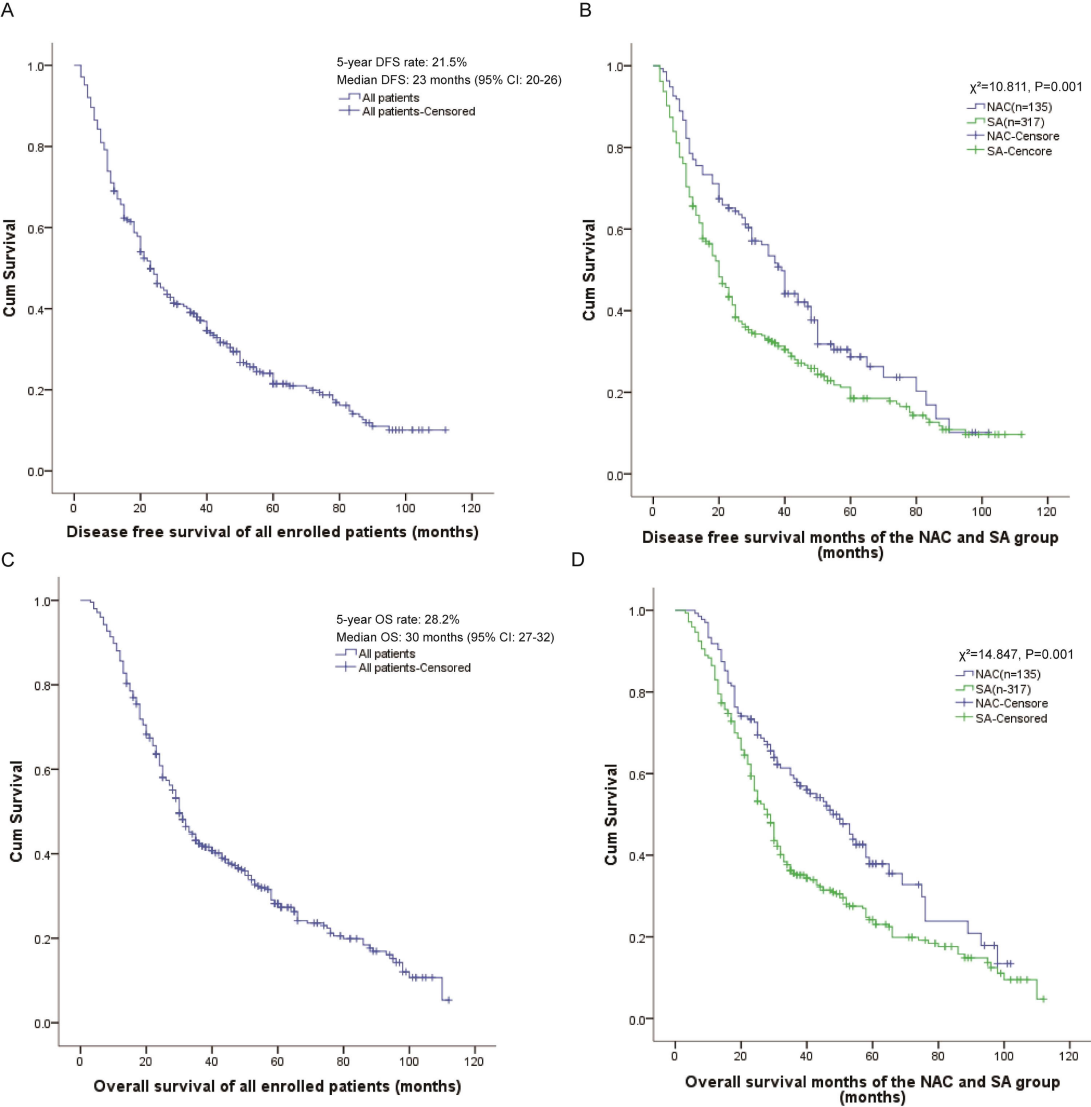
Evaluation of Disease-Free Survival (DFS) and Overall Survival (OS).

At the final follow-up, the overall cohort population demonstrated a 5-year DFS rate of 21.5% with a median DFS of 23 months (95% CI: 20-26) (**Figure 1A**). Comparative analysis revealed significant survival benefit in the NAC group, which exhibited superior 5-year DFS (28.7% vs. 18.5%; *P* = 0.001) and prolonged median DFS (39 months, 95% CI: 34–44 vs. 20 months, 95% CI: 17-23; χ²=10.811, *P* = 0.001) compared to the SA group (**Figure 1B**).

Similarly, survival analysis revealed a 5-year OS rate of 28.2% and median OS of 30 months (95% CI: 27-32) for the overall population (**Figure 1C**). Notably, the neoadjuvant chemotherapy arm maintained a significant survival advantage, demonstrating both higher 5-year OS (37.9% vs. 24.2%; *P* = 0.001) and extended median OS (48 months, 95% CI: 38–58 vs. 28 months, 95% CI: 25-31; χ²=14.847, *P* = 0.001) relative to SA group (**Figure 1D**).

### 3.3 Comparisons between pathological response, DFS and OS subgroups

**Figure 2:**
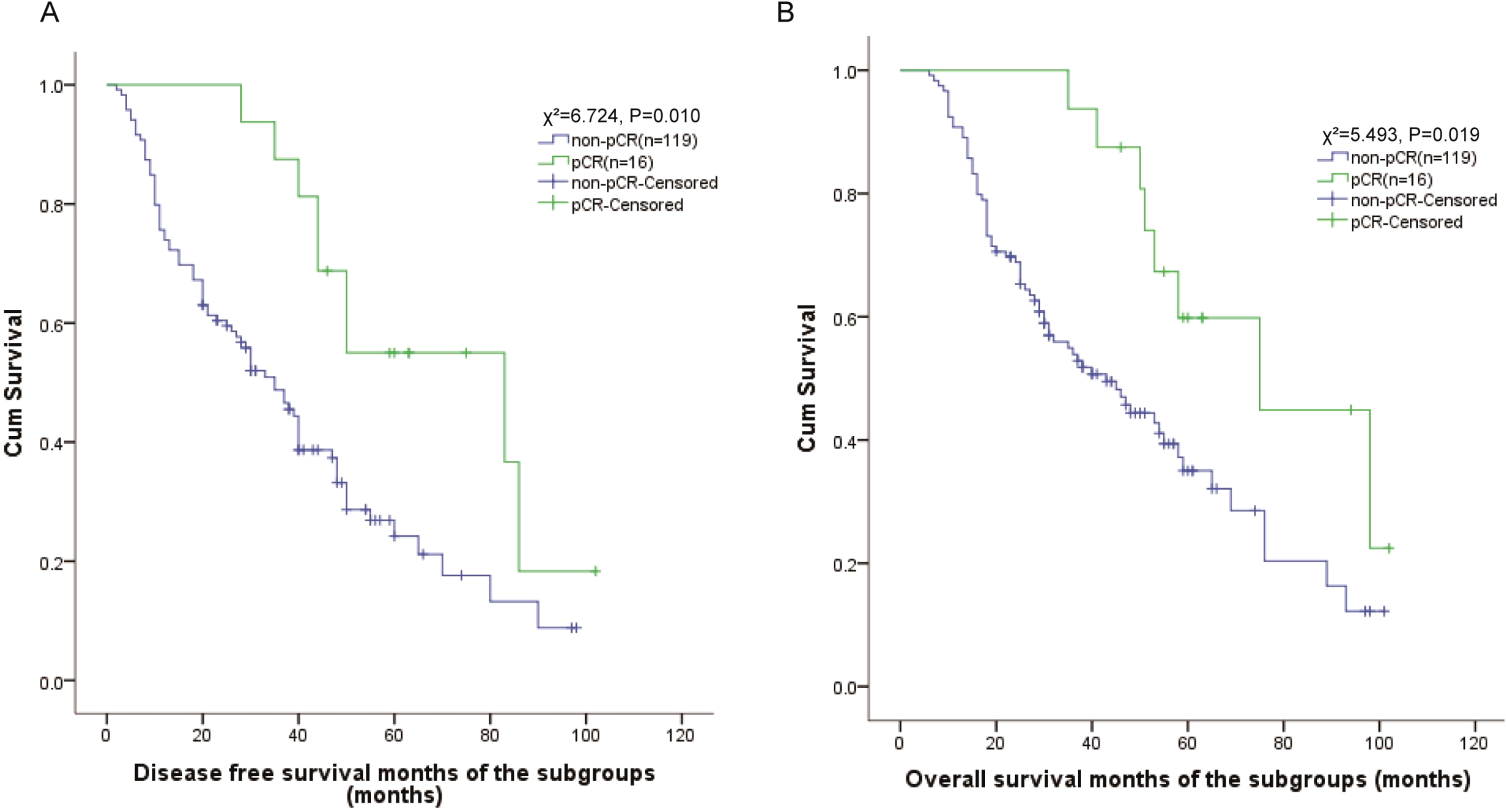
Comparison of DFS and OS among subgroups.

To further investigate prognostic factors in locally advanced ESCC, we performed subgroup analysis of 135 patients who received neoadjuvant chemotherapy. Pathological complete response (pCR) was achieved in 16 patients (11.9%). Kaplan-Meier survival analyses revealed significantly superior outcomes in pCR patients compared to non-pCR cases. Patients achieving pCR showed markedly improved DFS outcomes compared to non-pCR patients, with 5-year DFS rates of 55.0% vs. 18.5% (χ²=6.724, *P* = 0.010). The median DFS was 83 months (95% CI: 22-144) in pCR patients compared to 35 months (95% CI: 27-43) in non-pCR patients (**Figure 2A**).

Similarly, OS analysis demonstrated superior outcomes for pCR patients, with 5-year OS rates of 59.8% versus 35.0% in non-pCR patients (χ²=5.493, *P* = 0.019). The median OS was 75 months (95% CI: 40-111) in the pCR group compared to 43 months (95% CI: 30-56) in the non-pCR group (**Figure 2B**).

### 3.4 Univariable and multivariable Cox regression analysis of DFS and OS

Subsequently, univariate analyses were performed to identify variables potentially influencing DFS and OS. As shown in **Table 2**, neoadjuvant chemotherapy (χ²=10.811, *P* = 0.001), gender (χ²=4.843, *P* = 0.028), histologic grade (χ²=18.090, *P* < 0.001), ypT stage (χ²=22.136, *P* < 0.001), ypN stage (χ²=65.639, *P* < 0.001), vessel or nerve invasion (χ²=8.863, *P* = 0.003) and pCR (χ²=18.312, *P* < 0.001) as factors significantly associated with DFS. Similar variables were predictive of OS, including neoadjuvant chemotherapy (χ²=14.847, *P* = 0.001), gender (χ²=7.285, *P* = 0.007), histologic grade (χ²=19.750, *P* < 0.001), ypT stage (χ²=14.141, *P* < 0.001), ypN stage (χ²=70.291, *P* < 0.001), vessel or nerve invasion (χ²=8.224, *P* = 0.004), and pCR (χ²=5.493, *P* = 0.019) (**Table 2**).

**Table 2:** Univariable analysis of DFS and OS.

Characteristics	DFS		OS	
	χ^2^	P	χ^2^	P
Group	10.811	0.001	14.847	0.001
Gender	4.843	0.028	7.285	0.007
Age	0.558	0.455	0.067	0.795
Smoking	1.348	0.246	3.203	0.074
Alcohol Drinking	0.672	0.412	1.336	0.248
ECOG PS Score	0.000	0.984	0.058	0.809
Location	0.134	0.715	0.113	0.736
Histologic Grade	18.09	<0.001	19.750	<0.001
Surgical Approaches	0.144	0.705	0.426	0.514
ypT	18.312	<0.001	14.141	<0.001
ypN	65.639	<0.001	70.291	<0.001
Vessel or nerve invasion	8.863	0.003	8.224	0.004
pCR	6.724	0.01	5.493	0.019

To further investigate potential prognostic factors for DFS and OS, we conducted multivariate Cox regression analyses. The results identified the following as independent prognostic factors for DFS: neoadjuvant chemotherapy as a protective factor (HR: 0.752, 95% CI: 0.572–0.988, *P* = 0.041), and histologic grade (HR:0.637, 95% CI:0.512-0.792, *P* < 0.001 (G3 vs. G1)), ypT stage (HR: 2.242, 95% CI: 1.113-4.517, *P* = 0.024 (T3 vs. T0-1); HR: 4.599, 95% CI: 2.080-10.171, *P* < 0.001 (T4 vs. T0-1)), ypN stage (HR: 1.721, 95% CI: 1.330-2.227, *P* < 0.001 (N1 vs. N0); HR: 2.217, 95% CI: 1.629-3.019, *P* < 0.001 (N2 vs. N0); HR: 4.059, 95% CI: 1.303-3.558, *P* < 0.001 (N3 vs. N0)) as well as vessel or nerve invasion (HR: 2.153, 95% CI: 1.303–3.558, *P* = 0.003) as independent risk factors (**Table 3**). For OS, independent factors were neoadjuvant chemotherapy (HR: 0.699, 95% CI: 0.536–0.912, *P* = 0.008), histologic grade (HR:0.615, 95% CI:0.491-0.771, *P* < 0.001 (G3 vs. G1)), ypN stage (HR: 1.615, 95% CI: 1.236-2.109, *P* < 0.001 (N1 vs. N0); HR: 2.379, 95% CI: 1.758-3.268, *P* < 0.001 (N2 vs. N0); HR: 4.431, 95% CI: 2.797-7.017, *P* < 0.001 (N3 vs. N0)), vessel or nerve invasion (HR: 2.257, 95% CI: 1.343–3.793, *P* = 0.002), and pCR (HR: 0.451, 95% CI: 0.217–0.939, *P* = 0.033) (**Table 3**).

**Table 3:** Multivariable Cox regression analysis of DFS and OS.

Characteristics	DFS			OS		
	HR	95%CI	*P*	HR	95%CI	*P*
Group	0.752	0.572-0.988	0.041	0.699	0.536-0.912	0.008
Gender						
Age						
Location						
Histologic Grade	–	–	<0.001	–	–	<0.001
G1(ref.)	–	–	–	–	–	–
G2	1.672	0.530-5.278	0.381	1.807	0.571-5.714	0.314
G3	0.637	0.512-0.792	<0.001	0.615	0.491-0.771	<0.001
Surgical Approaches						
ypT	–	–	<0.001			
0-1(ref.)	–	–	–			
2	1.400	0.577-3.397	0.457			
3	2.242	1.113-4.517	0.024			
4	4.599	2.080-10.171	<0.001			
ypN	–	–	<0.001	–	–	<0.001
0(ref.)	–	–	–	–	–	–
1	1.721	1.330-2.227	<0.001	1.615	1.236-2.109	<0.001
2	2.217	1.629-3.019	<0.001	2.379	1.758-3.268	<0.001
3	4.059	1.303-3.558	<0.001	4.431	2.797-7.017	<0.001
Vessel or nerve invasion	2.153	1.303-3.558	0.003	2.257	1.343-3.793	0.002
pCR				0.451	0.217-0.939	0.033

Ref., Reference.

Collectively, both univariate and multivariate Cox regression analyses confirmed that neoadjuvant chemotherapy regimen, histologic grade, ypN stage, vessel or nerve invasion, and pCR were independent prognostic factors associated with DFS and OS.

## 4 Discussion

This retrospective study demonstrates that NAC followed by surgery significantly improves both DFS and OS compared to SA in patients with locally advanced ESCC. After PSM minimized selection bias, NAC was associated with superior 5-year DFS and OS. These findings align with prior evidence suggesting a survival benefit for NAC in ESCC (13–15).

Although the NCCN guidelines and other studies recommend neoadjuvant chemoradiotherapy (NCRT) as a standard for esophageal cancer (4, 14, 16), their applicability to ESCC is limited, as they are primarily derived from Western populations where adenocarcinoma predominates. This limitation is underscored by a Japanese study focusing on Asian ESCC patients, which found that NCRT did not confer a significant survival advantage over NAC and was associated with a higher incidence of treatment-related adverse events and postoperative complications (6). In this context, our findings provide novel and compelling insights into the ongoing debate regarding NAC as a viable alternative. Our study is distinctive in its focus on demonstrating the direct survival benefit of NAC over surgery alone in a real-world setting, thereby offering robust evidence for its clinical utility. Furthermore, our analysis incorporated detailed pathological assessments, including vessel or nerve invasion and CAP (Ryan) grading, which are critical prognostic factors often underreported in comparable studies.

A key innovation of this study lies in its comprehensive evaluation of pCR as a prognostic marker in NAC-treated ESCC patients. We not only confirmed that pCR was associated with significantly improved survival (17, 18) but also demonstrated its independent prognostic value in multivariate analysis. This finding is particularly relevant given the scarcity of large-scale studies examining pCR rates and their clinical implications specifically in NAC-treated ESCC, as most prior research has focused on NCRT (17). Our results suggest that pCR could serve as an early surrogate endpoint for treatment efficacy in NAC regimens, warranting further investigation in prospective trials. It is noteworthy that the pCR rate observed with NAC in this study was relatively low, a finding consistent with the results of the Japanese JCOG1109 trial (5%) and the study of Klevebro et al. (6, 19) but in stark contrast to the higher pCR rates typically achievable with NCRT (20). This discrepancy likely stems from fundamental differences in the mechanisms of action and pathological responses between the two modalities. NCRT combines the systemic effects of chemotherapy with the potent local intensification provided by radiotherapy, leading to more effective eradication of the primary tumor and consequently higher pCR rates. In contrast, NAC relies primarily on systemic chemotherapy, with its key objectives being the eradication of micrometastases and tumor downstaging. This may explain why NAC has a lower pCR compared to NCRT.

Additionally, our study advances the understanding of prognostic factors in NAC-treated ESCC by identifying vessel or nerve invasion as a strong independent predictor of poor outcomes. While previous studies have linked vessel or nerve invasion to adverse prognosis in ESCC (21–23), our work is among the few to validate its significance specifically in the NAC context, providing clinicians with valuable risk stratification tools. The inclusion of these pathological features in our multivariate model adds granularity to existing prognostic frameworks and may guide postoperative adjuvant therapy decisions.

While this real-world study provides valuable insights into the clinical outcomes of neoadjuvant chemotherapy in ESCC, several limitations should be acknowledged. First, while the retrospective design enhances the generalizability of our findings to real-world clinical practice, it may introduce inherent selection biases, despite our use of PSM to mitigate this concern. Furthermore, this study conducted numerous exploratory subgroup analyses to identify potential prognostic factors. However, these analyses were not adjusted for multiple comparisons, which inherently increases the risk of false-positive findings. Consequently, these results should be interpreted with caution and considered hypothesis-generating, requiring validation through future multi-center, prospective, randomized controlled trials. Second, as a single-center study, the generalizability of our findings may be limited by institutional-specific treatment protocols and patient populations. Although our current findings provide a theoretical basis for the application of NAC in ESCC, the inherent limitations of this single-center retrospective study necessitate further validation through multi-center, large-scale clinical trials. Third, despite effectively documenting survival endpoints, our study is subject to the inherent limitations of a retrospective design, including the lack of systematic data on treatment-related toxicity and patient quality of life. This gap prevents a comprehensive risk-benefit evaluation. It is therefore imperative that future prospective studies concurrently evaluate survival, toxicity, and patient-reported outcomes to guide optimal clinical decision-making. Finally, the evolving landscape of ESCC treatment, including emerging immunotherapies and targeted therapies, highlights the need for future prospective studies incorporating comprehensive biomarker analyses and standardized response assessment criteria to further optimize treatment strategies.

Our study not only reinforces the survival benefit of NAC in locally advanced ESCC but also introduces several novel contributions: (1) a PSM-balanced analysis confirming NAC’s efficacy versus surgery alone, (2) robust evidence for pCR as a prognostic marker in NAC-treated ESCC, and (3) validation of vessel or nerve invasion as key pathological risk factors. These findings underscore NAC as a valuable alternative to NCRT in select patients and point to a clear future direction: investigating biomarkers of chemotherapy sensitivity, such as ERCC1, could help identify patients most likely to derive significant benefit from NAC. The development of an integrated predictive model combining these biomarkers with clinical data is the logical next step towards tailoring neoadjuvant therapy, thereby optimizing outcomes and quality of life for individual patients.

With the evolving landscape of ESCC treatment, immunotherapy and targeted therapy have emerged as highly promising paradigms for future precision oncology. However, the relatively recent introduction of these modalities in our region means that robust long-term survival data are not yet available. Consequently, reliable comparisons regarding their efficacy and potential advantages over NAC remain elusive. In response to this knowledge gap, our center has initiated the systematic collection and follow-up of relevant data. Future work will focus on a more comprehensive and scientific analysis of the impact of NAC, NCRT, immunotherapy, and targeted therapy on survival outcomes in patients with ESCC.

## Data Availability

The raw data supporting the conclusions of this article will be made available by the authors, without undue reservation.
